# A Postauthorization Survey to Document the Therapeutic Management of Oxaliplatin as a First-Line Chemotherapy Regimen in South Africa in Patients with Metastatic Colorectal Cancer

**DOI:** 10.1155/2014/520701

**Published:** 2014-04-10

**Authors:** Lydia M. Dreosti, Alicia McMaster, Rashem Mothilal

**Affiliations:** ^1^Department of Medical Oncology, Faculty of Medical Sciences, University of Pretoria, Prinshof Campus, Steve Biko Academic Hospital, Dr. Savage Road, Pretoria 0001, South Africa; ^2^Medical Department, Sanofi South Africa, Midrand 1682, South Africa

## Abstract

Oxaliplatin is a standard first-line treatment for metastatic colorectal cancer. The objectives were to document the therapeutic management of oxaliplatin in South Africa, determine the incidence and severity of sensory neuropathy, and record the 2-year survival rate. Meccelox was a prospective, noncontrolled, open label, multicentre, observational survey of adult patients with stage IV metastatic colorectal cancer treated with oxaliplatin-based chemotherapeutic regimens. The study was conducted from August 2007 to November 2011 in 29 sites in South Africa by 66 participating treating physicians. Among the 195 enrolled patients, 61% were treated with FOLFOX regimen (5-fluorouracil/folinic acid plus oxaliplatin) for an average of 12 cycles and 32% patients were treated with XELOX (capecitabine plus oxaliplatin) for an average of 6–8 cycles, with the main reason for discontinuation being completion of the preplanned prescribed regimen. In Meccelox survey, 80% of patients were treated with intent of palliation. Overall 64% of patients reported symptoms of sensory neuropathy. The 2-year survival rate was 30%. *Conclusions*. Patients received a specified preplanned number of chemotherapy cycles rather than being treated until disease progression or toxicity. Both the incidence of neuropathy and the 2-year survival rate were less than previous reports.

## 1. Introduction

Colorectal cancer constitutes a major health problem worldwide representing the third most commonly diagnosed cancer in males and the second most common in females. Over 1.2 million new cancer cases and over 600,000 deaths occur globally each year [1]. Colorectal cancer can be considered as primarily a disease of the elderly, with more than 40% of cases occurring in patients over the age of 75 years [2].

In South Africa, incidence rates, after being standardized for age, are 8.6 per 100,000 males and 5.5 per 100,000 females, according to the country's national cancer registry (NCR) [3]. Age-standardised mortality rates in South Africa are 7.7 per 100,000 males and 4.5 per 100,000 females [4]. The most common site for metastases is the liver; synchronous liver metastases occur in 15 to 25% of newly diagnosed patients [5, 6]. Treating with both chemotherapy and surgical resection, overall 5-year survival rates of about 30–40% can now be achieved for patients with metastatic colorectal cancer [5].

Chemotherapy remains the cornerstone therapy for the treatment of metastatic colorectal cancer. First-line therapy for patients with metastatic colorectal cancer is rapidly evolving. Treatment regimens are moving away from fluoropyrimidine monotherapy to more complex and active combinations [3]. Various phase III trials have highlighted the efficacy of oxaliplatin. The response rate, progression-free survival, and quality of life are markedly increased with the addition of oxaliplatin to the De Gramont infusional fluorouracil/leucovorin (5FU/LV) regimen; (FOLFOX4) [7–10]. A pooled analysis of several randomized phase II and phase III trials investigating oxaliplatin in combination with capecitabine (XELOX) [11] showed similar progression-free survival and overall survival to FOLFOX [12]. These results therefore support the use of oxaliplatin as a standard first-line treatment of metastatic colorectal cancer [7].

The primary objective of this survey was to document the therapeutic use of oxaliplatin in the chemotherapy treatment of patients diagnosed with metastatic colorectal cancer. The main objectives were to record treatment intention, treatment regimens prescribed, treatment duration (number of cycles), and the reason for treatment discontinuation when patients are treated according to the treating physician's usual practice.

Secondary objectives included the following.

1. Documenting the incidence and severity of sensory neuropathy, since although oxaliplatin is generally well tolerated, cumulative peripheral sensory neuropathy characterized by a severe form of distal parasthesia may persist between cycles of treatment (dose-limiting toxicity) and cause functional impairment in activities of daily living [7].
2. Determination of the survival rate at 2 years and 5 years, of which only the former is presented here.

## 2. Methods

The Meccelox study was a prospective, noncontrolled, open-label, multicentre, observational postauthorization survey to document the therapeutic management of oxaliplatin as a first-line chemotherapy regimen in patients with metastatic colorectal cancer. The study was conducted from August 2007 to November 2011.

### 2.1. Patients

The study protocol was approved by the Institutional Review Boards of each site and a written informed consent was obtained from patients before study entry. Adult patients (≥18 years) with stage IV metastatic colorectal cancer were enrolled in the survey by 66 participating treating physicians from 29 medical centers across South Africa. Patients who had received adjuvant oxaliplatin within the previous 12 months were excluded from the survey, although those who had previously received adjuvant 5-FU or capecitabine as monotherapy were eligible for inclusion. For the dose and duration, treatments were to be guided by the prescribing information outlined in the registered prescribing information for oxaliplatin in South Africa.

### 2.2. Assessments

All patients were clinically evaluated at study entry including a full medical and surgical history. Patients were also evaluated for the presence of sensory neuropathy symptoms (graded according to the National Cancer Institute Common Terminology Criteria (NCI CTC) version 3 classification) (Table 1) [13], the Eastern cooperative oncology group (ECOG) performance status [14], and the use of any concomitant medications. Patients were given a diary to record symptoms and medications. Laboratory tests (haematology and clinical chemistry) were performed within 7 days and tumour markers within 14 days of the study entry visit. On day one of each chemotherapy treatment cycle, concomitant medications, laboratory tests including tumour markers, ECOG performance status, sensory neuropathy data, and any adverse events that occurred during the treatment cycle were recorded. NCI CTC version 3 was used to classify all serious adverse events, including sensory neuropathy. At the end of treatment, the total number of treatment cycles received, the reason for treatment discontinuation and planned subsequent treatment, sensory neuropathy data, and adverse events were documented. Finally sensory neuropathy symptoms, any study-treatment-related adverse events, any subsequent treatment, and any disease recurrence or death were recorded during patient visits or by telephone at 30 days after the end of treatment, at followup, and after 2 years. Data was collected by means of printed case report forms.

### 2.3. Statistical Analysis

Data from all the participating centers in South Africa were combined and treated as one dataset for the purposes of the analysis. The statistical analysis of the survey was of a descriptive nature where continuous variables were summarized by mean, median, standard deviation, minimum, and maximum values, and discrete variables were summarized by frequencies and percentages.

The efficacy endpoint of survival at two years was based on an intent-to-treat (ITT) analysis of all patients. The incidence of sensory neuropathy was defined as the number of new sensory neuropathy events per person time for those patients at risk.

All analyses were carried out on SAS, Release 9.2, run under Microsoft Windows for a personal computer.

## 3. Results 

### 3.1. Patients

The first patient was enrolled in August 2007, and the last patient out of the study was November 2011. The study was discontinued before the planned number of patients was recruited, due to low recruitment rate. A total of 195 patients (122 male) with a mean age of 60 years (ranging 24–88 years) were enrolled. Most patients were ECOG performance status 0-1. Previous or existing medical conditions were present in 71% of patients including 14% patients with neurological disorders, 4% with renal complaints, 6% with diabetes and/or hypertension, and 31% with cardiovascular conditions. The vast majority of patients (93%) had no symptoms of sensory neuropathy at study entry.

Chemotherapy had previously been administered to 20% patients, mainly in the adjuvant setting (74% of them) and 90% of patients had prior surgery pertaining to colorectal cancer (Table 1).

In the present study, 81% of patients had poorly or moderately differentiated histology at survey entry (Table 1).

### 3.2. Treatment

In 80% of patients the treatment intent was palliative, while 20% received neoadjuvant chemotherapy in preparation for liver metastasectomy. The planned treatment regimen was the FOLFOX regimen (infused 5FU/LV and oxaliplatin in a 14 day cycle) in 118 (61%) patients and the XELOX regimen (capecitabine and oxaliplatin in a 14 day cycle) in 62 (32%) patients [8, 9, 11]. The remaining 15 patients were planned to be treated with other regimens which are generally considered nonstandard of care. No patients received biological agents as part of the treatment for metastatic disease which at the time of the study were newly introduced to South Africa and not funded because of cost. The average duration of treatment for patients receiving the FOLFOX regimen (every 2 weeks) and XELOX regimen (every 3 weeks) were 12 cycles and 6–8 cycles, respectively (Table 2). This accounts for approximately 6 months of treatment in both regimens. Fifty-nine percent of patients were taking concomitant medications. In the FOLFOX regimen 2 patients continued beyond 14 cycles of treatment, with 1 patient continuing for 22 cycles. In the XELOX regimen, 3 patients continued treatment beyond 8 cycles with 1 patient continuing for 16 cycles.

### 3.3. Treatment Response

At least 50% of the patients at the end of oxaliplatin treatment had either a complete or partial response or stable disease, whereas only 18% of patients are known to have experienced disease progression during this time (Table 2). The response to treatment was not recorded in 62 patients; 13 of whom withdrew consent and treatment was withdrawn in another 13 at the physician's decision. It is unclear why the response was recorded as unknown in the remaining 46 patients. A total of 51% of patients had progressive disease during the 2-year survey analysis. At the end of 6 months of therapy 46% of patients discontinued treatment (Table 3). Twenty-four percent of patients are known to have had subsequent chemotherapy after the 30 day posttreatment assessment. The majority of patients had no change in their ECOG performance status during the survey irrespective of the number of chemotherapy cycles administered.

### 3.4. Survival at 2 Years

Ninety-four percent of the patients were alive at 30 days after oxaliplatin treatment discontinuation. At the two-year follow-up point, 30% of patients were alive (Table 4), including 3 out of 10 patients under 40 years of age. Of the patients still alive at 2 years, more than half (59%) of these patients had experienced a relapse during the 2-year follow-up period. The most frequent cause of death was colorectal cancer (Table 5).

Disease progression was experienced in 51% of the total cohort during the 2-year follow-up.

### 3.5. Safety

The most frequent adverse event was sensory neuropathy, occurring altogether in 64% of patients (Table 6). The incidence of sensory neuropathy symptoms increased with each cycle of chemotherapy. A greater percentage of patients receiving the FOLFOX regimen had sensory neuropathy symptoms compared to those receiving the XELOX regimen (Table 7). During oxaliplatin treatment, 45% of the sample's patients had symptoms of sensory neuropathy, mostly mild symptoms (Grade 1), at the time of the two year follow-up, the total percentage of patients with sensory neuropathy had dropped to 5% (Table 8).

A serious adverse event is an adverse event that meets one or more of the following criteria: results in death, is life-threatening, requires in-patient hospitalization or prolongation of existing hospitalization, results in persistent or significant disability or incapacity, or is considered to be medically important by the investigator.

## 4. Discussion

The primary focus of the Meccelox study was to record how patients with metastatic colorectal cancer are treated with chemotherapy regimens including oxaliplatin in a* real-life* setting. Since oxaliplatin is considered a first-line therapy for these patients, it is important to gauge how otherwise unconsidered factors may contribute to its efficacy and toxicity in practice.

### 4.1. Primary Objective

Our main objective was to document treatment intention, treatment regimens prescribed and their duration, and the reason for treatment discontinuation.

In the survey's sample, 80% of patients were being treated for the purpose of palliation. Most patients were on the XELOX or FOLFOX regimens and the main reason recorded for discontinuing treatment was due to completion of the preplanned number of cycles of the prescribed regimen. Indeed, the findings clearly indicate that treatment is generally stopped after 6 to 8 cycles with XELOX treatment and after 12 cycles with FOLFOX treatment (Table 7).

Normally palliative treatment is continued until the patient experiences progressive disease when on the treatment or the patient develops toxicity from the drug [15]. The findings show that this line of therapy is not being practiced in South Africa; patients receive only a specified preplanned number of cycles of chemotherapy. In most phase III studies treatment was continued until disease progression or was stopped due to toxicity. It was not stopped after administering a prespecified number of cycles. In the NO 16966 trial the median number of cycles of FOLFOX was 11 (range 1–24) and the median number of cycles for XELOX was 7 (range 1–18). In the last few years there has been a shift in treatment strategy from prescribing specific successful lines of therapy until disease progression to that of “continuum of care,” in which chemotherapy is tailored to the individual and may be discontinued so that other chemotherapy, maintenance therapy, therapy breaks, and surgery can be taken with the goal of improving survival and quality of life [15]. This approach, as before, is always mindful of toxicity. It is possible that a reason for treatment termination in this survey could be due to a perceived risk of toxicity, particularly if treatment continues beyond 6 months. After discontinuation of oxaliplatin treatment only 24% of patients were known to have received subsequent lines of chemotherapy treatment. This highlights the fact that premature cessation of treatment should be addressed with oncologists in South Africa. The local health sector in this country has become extremely cost sensitive with the majority of the population relying on government funding. It is therefore likely that there is an impact of medical funder policies in the private sector on treatment management where funding is only ensured for 6 months.

### 4.2. Two-Year Survival

The reported median survival with 5-fluorouracil and leucovorin treatment for colorectal carcinoma ranges around 14 months, with only 21% of these patients alive at 2 years [9, 16]. The addition of oxaliplatin and availability of second-line therapies improve the median overall survival and have been reported for most trials as 20 months or more [7]. In our study we recorded that only 30% of the total cohort were alive at 2 years. Even accounting for a few patients lost to followup or with missing status reports, this is similar to that reported for 5-fluorouracil and leucovorin therapy alone. Funding issues and other real-life practicalities that influence treatment duration and percentage of patients receiving second-line treatment may well result in turn in poorer outcomes. Another possible reason could be the aggressiveness of the disease.

Although this secondary endpoint was not analyzed separately for each regimen, several recent meta-analyses and phase III trials have given strong evidence to indicate that the overall survival and progression-free survival are similar between XELOX and FOLFOX type regimens [12].

### 4.3. Safety Data

Mild gastrointestinal and haematological side effects are commonly associated with oxaliplatin when combined with 5-FU, with the principle toxicities being neurotoxicity and neutropenia [17]. Acute neurotoxicity (paraesthesia or dysaesthesia), although frequently seen, is generally transient and mild [15].

Chronic cumulative neurotoxicity develops in most patients. After several cycles of oxaliplatin therapy, a late-onset cumulative sensory neuropathy may occur [17]. Significant neurotoxicity causing functional impairment (Grade 3/4 toxicity) is reported in most trials at 15%–17% of patients [18]. In the Meccelox survey grade 3/4 neurotoxicity was seen in 4.9% of the neuropathy episodes reported.

In the Meccelox survey the most frequently reported adverse effect was sensory neuropathy (all grades) which was reported in 64% of patients.

At survey entry 7% of patients had sensory neuropathy present. This could either be due to previously administered chemotherapy or other comorbidities such as diabetes documented in some patients. At all assessment stages we found the vast majority of neuropathic symptoms to be mild (grade 1 or grade 2). We observed an increased proportion of patients reporting sensory neuropathy symptoms with increasing number of cycles. This adverse effect typically improves rapidly with the discontinuation of oxaliplatin treatment [19] and in our study we found little evidence of sensory neuropathy at the 2-year followup.

It is interesting to note that the patients who received the XELOX regimen experienced less sensory neuropathy per cycle than the patients treated with the FOLFOX regimen. XELOX is known to have a higher incidence of hand-and-foot syndrome, but previous studies have reported a similar incidence of sensory neuropathy when compared to FOLFOX [20]; grades 3 and 4 neurotoxicity are being reported as 17% for both regimens. However, other factors such as physician preference may have also played a role in our sample, where almost twice as many patients were treated with FOLFOX compared to XELOX. Returning to the issue of funding, XELOX has been shown to have slightly higher direct costs to FOLFOX but far less indirect costs. Furthermore XELOX may be more convenient and easier for both the patient and physician, since capecitabine is taken orally without the substantial cost and difficulties of placing a central vein catheter, and oxaliplatin is administered every 3 weeks in comparison to the two weekly administrations with FOLFOX [11]. These reduced costs, along with the possibility of a reduction in neuropathic side effects, may mean that medical insurance coverage could fund a greater number of cycles for patients who require them rather than deciding in advance on the duration of treatment.

Good clinical practice recommends monitoring of disease progression at regular intervals throughout treatment laboratory tests such as full blood count and urea and electrolytes every week and liver function every second visit were either not carried out or were poorly reported in the survey. If the former was the case this further emphasizes factors that need to be considered in a real-life setting, for example, availability of professional personnel to carry out the tests, time constraints, and equipment availability and cost. Similarly good clinical practice recommends that the response to treatment be assessed not only during treatment but also at the end of treatment. Whilst it is possible that the reluctance of funders to pay for more cycles of therapy may have been a factor for not measuring response at the end of treatment, of concern is the prescription of a prespecified number of cycles.

## 5. Conclusions

Oxaliplatin has allowed metastatic colorectal cancer to become a chronic, treatable disease. The findings from this survey have highlighted factors that need to be considered by oncologists and medical coverage providers when prescribing these regimens in a real-life setting. Treatment was only administered for a specified preplanned number of cycles. The 2-year survival rate of 30% is less than reported in previous studies, where oxaliplatin-based chemotherapy was used in metastatic colorectal cancer and where second-line therapies were more systematically used. Sensory neuropathy was experienced by 64% of the study population with grade 1/2 neuropathy accounting for 94.5% of neuropathy episodes.

## Figures and Tables

**Table 1:** Patient demographics and baseline clinical characteristics.

Characteristic	Number of patients (%)
Sex	
Male	122 (63)
Female	73 (37)
Age (years)	
Median	62
Mean	60
Range	24–88
ECOG performance status	
Grade 0	63 (32)
Grade 1	86 (44)
Grade 2	10 (5)
Grade 3	2 (1)
Grade 4	0
Unknown	34 (17)
Neuropathy grade	
Grade 0	182 (93)
Grade 1	12 (6)
Grade 2	0
Grade 3	1 (1)
Grade 4	0
Initial pathology type	
Adenocarcinoma	192 (99)
Mucinous colloid	54 (28)
Signet ring	3 (2)
Other*	3 (2)
Sites of metastases	
Liver	139 (71)
Lung	49 (25)
Lymph node	47 (24)
Brain	1 (0.5)
Bone	8 (4)
Peritoneum	20 (10)
Other	35 (18)
Histology	
Poorly differentiated	20 (10)
Moderately differentiated	137 (70)
Well differentiated	9 (5)
Unknown	29 (15)
*Tumour markers *	
CEA	
Within range 0–5 ng/mL	38 (20)
Above range	98 (50)
Unknown	59 (30)
CA	
Within range 0–37 u/mL	41 (21)
Above range	55 (28)
Unknown	99 (51)
Past or present medical history excluding colorectal cancer	
Negative	57 (29)
Positive	138 (71)
Surgical history relevant to colorectal cancer	
Negative	20 (10)
Positive	175 (90)
Previous exposure to chemotherapy for colorectal cancer	
Negative	157 (80)
Positive^¶^	38 (20)
Single	16 (9)
Mayo	14 (7)
Other	8 (4)

The percentages are calculated out of the total survey's cohort of 195 patients. Totals are occasionally above 195 due to multiple reporting for example initial pathology type and number of metastases.

ECOG: Eastern cooperative oncology group; CEA: carcinoembryonic antigen; CA: cancer antigen.

*Including infiltrated lymph around veins, moderate to poor differentiated, nonspecified.

Laboratory tests included full blood count, liver function test, and urea and electrolytes, of which only a few are listed here.

^¶^“Single” agents included 5-FU, fluroblastin, or Xeloda; “Mayo” included 5-FU and leucovorin or 5-FU and Rescuvolin or ABIC fluorouracil and Isovorin; “Other” included FOLFIRI, FOLFIRI with bevacizumab, FOLFIRI followed by Capecitabine, XELOX, and FOLFIRI with mitomycin, Tomudex, and capecitabine. Of those patients who received chemotherapy for colorectal cancer prior to this study, 28 (74%) patients received the chemotherapy in the adjuvant setting.

**Table 2:** Best response at the end of oxaliplatin treatment.

Response	Number (%) of patients
Complete response	11 (6)
Partial response	47 (24)
Stable disease	40 (20)
Progressive disease	35 (18)
Unknown	62 (32)

The percentage is calculated out of 195, the total number of patients in the survey's cohort.

**Table 3:** Reasons for discontinuation of treatment with oxaliplatin.

Reason	Number (%) patients
Completed the prescribed regimen	90 (46)
Disease relapse/recurrence	31 (16)
Adverse events	24 (12)
Death	12 (6)
Patient withdrew consent	3 (2)
Patient withdrew treatment consent only	10 (5)
Physician's decision to discontinue treatment	13 (7)
Other	12 (6)

The percentage is calculated out of 195, the total number of patients in the survey's cohort.

**Table 4:** Survival status at the 2-year followup.

	Number (%) of patients
Alive	58 (29.7)
Dead	126 (64.6)
Lost to followup	4 (2.1)
Missing status reports	7 (3.6)

The percentage is calculated out of 195, the total number of patients in the survey's cohort.

**Table 5:** Causes of death at 30 days after oxaliplatin treatment discontinuation.

Cause of death	Number of patients (%)
Metastatic colorectal cancer	8 (4.1)
Pneumonia with septicaemia	1 (0.5)
Neutropenic fever	1 (0.5)
Haemorrhage of unknown origin	1 (0.5)
Toxicity related to chemotherapy	1 (0.5)
Total	**12 (6)**

The percentage is calculated out of 195, the total number of patients in the survey's cohort.

**Table 6:** Adverse events.

Event	Number (%) of patients	Serious or not	Number (%)
Sensory neuropathy	124 (63.6)	No Yes	122 (62.6) 2 (1.0)
Intermittent neutropenia	58 (29.8)	No Yes	53 (27.2) 5 (2.6)
Intermittent thrombocytopenia	30 (15.4)	No Yes	30 (15.4) —
Intermittent anemia	39 (20.0)	No Yes	36 (18.5) 3 (1.5)
Intermittent nausea	76 (39.0)	No Yes	71 (36.4) 5 (2.6)
Intermittent diarrhoea	78 (40.0)	No Yes	66 (33.8) 12 (6.2)
Intermittent vomiting	29 (14.9)	No Yes	23 (11.8) 6 (3.1)
Stomatitis	28 (14.4)	No Yes	28 (14.4) —
Skin urticaria	8 (4.1)	No Yes	8 (4.1) —
Hand-foot syndrome	20 (10.3)	No Yes	20 (10.3) —
Alopecia	13 (6.7)	No Yes	13 (6.7) —
Allergy	10 (5.1)	No Yes	9 (4.6) 1 (0.5)
Thrombosis/phlebitis	3 (1.5)	No Yes	1 (0.5) 2 (1.0)
Neutropenia with fever	2 (1.0)	No Yes	1 (0.5) 1 (0.5)
Infection	20 (10.3)	No Yes	15 (7.7) 5 (2.6)

Percentages were calculated out of the sample size of 195 patients and do not add up to 100% due to multiple reporting.

**Table 7:** Presence of neuropathy on the FOLFOX and XELOX regimens.

Cycle	FOLFOX grade neuropathy per cycle (%)	XELOX grade neuropathy per cycle (%)	Total grade neuropathy per cycle				
	All	All	All	Grade 1	Grade 2	Grade 3	Grade 4
Cycle 1	5/118 (4.2)	4/62 (6.5)	12/195	9	2	1	—
Cycle 2	30/114 (26.3)	13/59 (22.0)	52/185	46	5	1	—
Cycle 3	47/109 (43.1)	15/52 (28.8)	69/170	60	8	1	—
Cycle 4	53/104 (51)	13/48 (27.1)	73/161	60	11	2	—
Cycle 5	55/99 (55.6)	13/42 (31)	73/149	58	14	1	—
Cycle 6	53/96 (55.2)	10/34 (29.4)	69/138	49	14	4	2
Cycle 7	51/84 (60.7)	6/21 (28.6)	58/109	45	11	2	—
Cycle 8	49/80 (61.3)	6/15 (40)	57/99	45	11	1	—
Cycle 9	50/76 (65.8)	2/3 (66.7)	53/83	43	9	1	—
Cycle 10	43/70 (61.4)	0/2 (0)	44/75	32	9	3	—
Cycle 11	47/66 (71.2)	2/2 (100)	50/70	30	17	3	—
Cycle 12	42/60 (70)	2/2 (100)	46/64	26	11	9	—
Cycle 13	5/9 (55.6)	1/2 (50)	6/11	5	1	—	—
Cycle 14	3/7 (42.9)	1/2 (50)	5/9	3	—	2	—
Cycle 15	1/2 (50)	1/1 (100)	2/3	1	1	—	—
Cycle 16	2/2 (100)	0/1 (0)	2/3	—	2	—	—
Cycle 17	2/2 (100)		2/2	—	2	—	—
Cycle 18	2/2 (100)		2/2	—	2	—	—
Cycle 19	1/1 (100)		1/1	—	1	—	—
Cycle 20	1/1 (100)		1/1	—	1	—	—
Cycle 21	1/1 (100)		1/1	—	1	—	—
Cycle 22	1/1 (100)		1/1	—	1	—	—
Total	**544**	**89**	**679 (100%)**	**512 (75%)**	**134 (19.7%)**	**31 (4.6%)**	**2 (0.3%)**

**Table 8:** Presence of neuropathy.

	Number (%) of patients with neuropathy symptoms				
	Total	1	2	3	4
30 days after oxaliplatin treatment discontinuation	87 (48)	46 (25)	18 (10)	20 (11)	2 (1)
2-year followup	9 (5)	5 (5)	4 (4)	—	—

Percentages are calculated out of 195, the total number of patients in the sample. One of the patients with neuropathy symptoms at 30 days after treatment had an unknown symptom grade.

Neuropathy grade refers to the Sanofi-Aventis oncology classification for any neuropathy symptoms: (1) paraesthesia or dysaesthesia not interfering with function; (2) paraesthesia or dysaesthesia interfering with function, but does not interfere with activities of daily living; (3) paraesthesia or dysaesthesia with pain or function impairment that interferes with activities of daily living; (4) persistent paraesthesia or dysaesthesia that is disabling or life-threatening.
